# Coping with in-locus factors and systemic contradictions affecting antibiotic prescription and dispensing practices in primary care–A qualitative One Health study in Brazil

**DOI:** 10.1371/journal.pone.0280575

**Published:** 2023-01-20

**Authors:** Roberto Rubem da Silva-Brandao, Sandi Michele de Oliveira, Juliana Silva Correa, Luiz Felipe Zago, Lislaine Aparecida Fracolli, Maria Clara Padoveze, Gloria Cristina Cordoba Currea

**Affiliations:** 1 Nursing School, University of São Paulo, São Paulo, Brazil; 2 School of Public Health, University of São Paulo, São Paulo, Brazil; 3 Section of General Practice, Institute of Public Health, Faculty of Health and Medical Sciences University of Copenhagen, Copenhagen, Denmark; 4 Antimicrobial Research Unit, School of Health Sciences, University of KwaZulu-Natal, Durban, South Africa; Universidade Catolica Portuguesa, PORTUGAL

## Abstract

Antimicrobial resistance (AMR) is an increasing threat to global health. The risks and sanitary consequences of AMR are disproportionately experienced by those living in Low- and Middle-Income Countries (LMICs). While addressing antibiotic use has largely been documented in hospital settings, the understanding of social drivers affecting antibiotic prescribing and dispensing practices in the context of human and animal health in primary care (PC) in LMICs remains extremely limited. We seek to explore how in-locus and multi-level social factors influence antibiotic prescriptions and dispensing practices in the context of human and animal health in primary care in Brazil. This is a baseline qualitative One Health study; semi-structured interviews and field observations were undertaken in primary care sites located in a socioeconomically vulnerable area in the city of São Paulo, the most populated city of Brazil. Twenty-five human and animal healthcare professionals (HP) were purposely sampled. Interview data were subject to thematic analysis. Three overlapping social drivers were identified across HPs’ discourses: individual and behavioral challenges; relational and contextual factors influencing the overprescription of antibiotics (AB); and structural barriers and systemic contradictions in the health system. As a result of the interaction between multilevel in-locus and structural and contextual factors, HPs experience contextual and territorial challenges that directly influence their risk perception, diagnosis, use of laboratorial and image exams, time and decision to undergo treatment, choice of AB and strategies in coping with AB prescriptions. Additionally, in-locus factors influencing antibiotic prescriptions and dispensing practices are intertwined with individual accounts of risk management, systemic contradictions and ambivalences in the national health system. Our findings suggest interventions tackling AB use and AMR in Brazil should consider the social context, the complex health system structure and current integrated programs and services in PC.

## Introduction

Antimicrobial resistance (AMR) is an increasing threat to global health and economies. The risks and sanitary consequences of AMR are disproportionately experienced by those living in Low- and Middle-Income Countries (LMICs), particularly because of socioeconomic factors such as material deprivation, sanitation infrastructure and lack of access to medications and to human and animal health systems [1]. A growing body of literature has documented the vital role of social science in understanding and intervening on key drivers of AMR, particularly on antibiotic (AB) use [2, 3]. While addressing AB use has been often documented in hospital settings over the last decade [4, 5], the understanding of social drivers affecting AB prescribing and dispensing practices in the context of human and animal health in primary care (PC) of LMIC remains extremely limited.

Within LMIC contexts, scholarly works have documented the economic disparities affecting access to AB and health resources, including the awareness of health professionals (HPs) about the risks of AB overprescription and AMR [6–8]. Noticing that high-income countries’ prescribers and dispensers of AB have largely been subject to AMR stewardship practices and health interventions in PC, the vast majority of LMICs still struggle to implement interventions and operational National Action Plans (NAPs) to respond to AMR [8]. While the literature has identified social and political drivers influencing the AMR global threat [3, 9, 10], empirical studies have, however, focused on understanding HPs’ behavior, individual prescribing practices and knowledge related to AB use and AMR [3, 11]. Additionally, the problem of AB overprescription and AMR has largely been studied without approaching the human and animal health together in qualitative studies; most of LMIC study settings have not documented comprehensive social science multifactorial and multilevel qualitative data regarding the problem of AMR in socioeconomically deprived contexts.

More importantly, there are contentious gaps in the literature of social drivers affecting AB use and AMR globally, and particularly in LMICs: (i) while economic deprivation has largely been approached as a major factor affecting access to adequate healthcare and resources [2, 8], it has been approached hegemonically as a linear factor separated from the analysis of health system structures; (ii) as studies have emphasized the central role of HPs’ practices in AMR stewardship, scholarly works have provided little evidence on how HPs’ roles and conduct are entangled by health systems structure and territorial demands. Additionally, the challenges HPs and health services face with concurrent other health demands/issues are little explored in the literature about AMR in PC [12]; (iii) considering that studies have approached social drivers as mediators, determinants and/or subject to political actions amid the global threat of AMR [2, 7, 8], qualitative studies have not extensively explored how social factors are intertwined, overlapped and/or interrelated within the HPs’ practices toward AMR stewardship in PC; finally, (iv) while a growing body of literature has asserted the need for health interventions on AMR to be sensitive to local contexts and health systems functioning [7, 13, 14], studies have not thoroughly explored how PC services are used and distributed in the LMIC’s local communities, affecting health demands and responses to AMR.

Seeking to shed light on these issues, this article explores the social determinants and drivers related to inappropriate prescriptions of AB, presenting a framework to discuss the social implications of this increasing public health threat in the context of PC of human and animal health in the city of São Paulo, Brazil. We adopt a non-linear relational perspective between social drivers affecting AMR and PC by discussing in-locus factors that may inform inappropriate antibiotic prescriptions and reveal systemic contradictions in the Brazilian National Health System (SUS). This approach allows us not only to identify but further explore the relationships between major social determinants and drivers of the problem, while setting priority areas for possible health interventions.

This study considers the ramifications of the One Health approach to AMR research. One Health refers to a collaborative, multisectoral and transdisciplinary approach to achieve optimal health and well-being outcomes by recognizing the interconnections between people, animals, plants and their shared environment [15]. We utilize this theoretical-methodological perspective that articulates the One Health approach and the social production of the AMR phenomenon while studying the practices of prescribing and dispensing AB by human and animal health professionals in PC. We mainly explore the capillary of the SUS in PC, a human universal and free of charge healthcare system, through its territorial organization, and a public Veterinary institution in the periphery of São Paulo. To our knowledge, this is the first time a study reports a One Health perspective exploring animal and human HPs’ prescribing and dispensing practices in PC considering in-locus and multi-level factors influencing antibiotic prescriptions and dispensing practices within a LMIC context.

## Methods

We followed the Standards for reporting qualitative research in conducting and describing this study [16].

### Context

This study is part of a large One Health initiative studying AB use and AMR in Brazil exploring three major axes: (i) AB use from the point of view of health services’ users; (ii) the AB prescribing and dispensing practices in primary care, whose major results are presented in this article; and (iii) the process of building the political agenda to fight AMR in Brazil. The three areas worked in tandem, with researchers exchanging data, conclusions, and reflections throughout the entire research protocol. Although the three axes were developed and conducted with complete autonomy, our results benefit from the reflections of each perspective.

The first author (cis-male, self-identified) conducted all the interviews. The interviewer is a HP (licensed nutritionist) and social scientist and has experience with qualitative research methods and sociological theory, being a PhD candidate in Public Health at the time of the study. Additionally, the interviewer is a SUS user, very familiar with PC service and the role of each HP.

### Study design

We utilized the One Health theoretical and methodological approach in order to explore the social production of the AMR phenomenon while studying the practices of prescribing and dispensing AB by human and animal health professionals in PC.

The One Health approach is a holistic perspective based on the acknowledgement that the common environment in which humans and animals interact results in shared health outcomes such as the development of AMR [17]. For example, inappropriate use of antibiotics in humans and animals is one of the main reasons that antibiotics are becoming ineffective, thus threatening patient security and quality treatment. Understanding the different levels of interactions regarding antibiotic use in humans and animals is paramount for developing effective and long-lasting solutions for the benefit of all. During the last decade the One Health approach has gained political recognition as the best strategy to bring about effective and sustainable solutions to curb AMR [18].

In-depth semi-structured interviews and contextual observations were undertaken with HPs in six urban health units in a periphery area in southern São Paulo, Brazil. We aimed to understand HP’s awareness and practices regarding prescribing and dispensing AB, as well as the relationship between contextual factors, human and animal health practices and antimicrobial use in PC.

### Settings and recruitment

The sampling strategy aimed to include those registered to prescribe and/or dispense antimicrobials: doctors, nurses, dentists, pharmacists, and veterinarians. Study participants who were AB prescribers worked in a Primary Care Unit (PCU) for human health (nurses, pharmacist and general practitioners [GP]) chosen prior to the initiation of the study, a specialized public dentistry service that offers primary and secondary health care (dentists) that function in the PCU, and a Public Veterinary Hospital that provides services from primary to tertiary health care levels (Veterinarians). Pharmacists were interviewed from three additional Ambulatory Health Services (AHS) in the territory that provides human secondary level health care; AHS were included in the study because they attended similar cases as in PC and were responsible for providing secondary care in different parts of the territory, in addition to its diverse services and demands that were very informative in the context of practices in the territory. We did not seek to compare health settings in regarding to AB dispensing and use practices; rather, the health units reflect the closest combination of services that patients use in the local area of study. Our primary contact with the PCU’s local manager was established by one of our study coordinators, who had previously worked in research with the local manager; we researched the contact information of the other health settings’ managers and approached them directly to arrange a meeting to present the study. Before recruitment, the interviewer, the first author, presented himself and the study (scope, aims, goals and scientific partnerships involved in the research) to all GPs, nurses and pharmacist located in the PCU, and to both dentistry and veterinary managers. In these occasions, the interviewer acknowledged his role as a research assistant of the study, the institutions and investigators involved in the study and the source of funding, highlighting the scientific orientation of the study. In addition, the interviewer pointed out that the study aim was not to assess or judge any professional conduct and that the HPs’ experiences and views were very much appreciated throughout the entire research process. HPs also were informed of the potential benefits of contributing to the research on AMR in Brazil, which may orient future local health interventions. In all presentations, HPs and local managers were enthusiastic about the study and expressed the importance of exploring the theme of AMR in PC. All queries from HPs and local managers were promptly answered in person or *via* e-mail prior to recruitment. The interviewer did not know any personnel working in the health units before study commencement. There was no conflict of interest between the interviewer and the HPs and their workplaces.

Inclusion criteria for the study required practitioners to be registered prescribers and/or dispensers of antimicrobial medicines in the local public services. Experience working in PC for less than a year constituted an exclusion criterion in this study. We employed few inclusion and exclusion criteria in order to *reflect* the local reality of the health settings in a vulnerable area of São Paulo. Participants were approached both face-to-face and *via* telephone. HPs working in the human PCU and the Public Veterinary Hospital were approached face-to-face after study presentation in a regular weekly meeting in the Unit. Pharmacists working in AHS were approached *via* telephone; their institutional telephone numbers were provided by one pharmacist (study participant) working in the PCU. Potential participants were approached to participate in the study through a written informed consent process which included permission to conduct, record and transcribe interviews.

Interviews comprised the primary method of data collection; moreover, researchers also carried out contextual observations in order to familiarize themselves with the local health setting and community. In this way, prior to the interviews, supervised visits to the health settings and local community were realized in presence of either HP and/or managers. At this phase, field notes were mainly taken to adjust questions in the interview guide to better reflect the local reality. During fieldwork, field notes were immediately taken after each interview, providing additional insights and information derived from interviews that were also subject to analysis. None of the HPs we approached refused to participate in the study.

### Sampling strategy

The research was conducted in partnership with local health institutions that manage the public and free-of-charge health services. Participants were selected using purposive sampling. The criterion sampling strategy has been designed to reflect the diversity of HPs attending a heterogeneous population of patients within pragmatic limits: different professional roles that each HP performed, human and animal health settings that function in different parts of the same district; we made efforts to achieve gender and age balance among the interviewees.

### Ethical issues pertaining to human subjects

This research was conducted following the Declaration of Helsinki on studies with humans. This study was submitted and approved by the Ethics Review Committees from the National Commission on Ethics in Research (CONEP) in Brazil, under number 42442921.7.0000.5392. All study participants provided their written informed consent for inclusion and audio recording before they participated in the study.

### Data collection procedure

The researcher arranged for the interview to be conducted at a convenient time for the participant. After providing informed consent, participants were interviewed individually, face-to-face, with no third parties present. All interviews occurred in the HPs’ working hours in the health units and lasted an hour, on average. All interviews were conducted in Portuguese. Data collection took place between August 2021 to February 2022.

### Data collection instruments and technologies

Year of birth, sex and occupational status were collected using self-reports. The development of the interview guide arose as a research team effort. External consultants also provided feedback on the interview guide; meetings were conducted with specialists in veterinary, general practice and pharmacy. A simulation interview was conducted with a registered GP that worked in the SUS prior to the recruitment of participants. In this way, external contributions provided feedback to develop core set of areas and test the clarity and structure of the interview guide. Each question was also checked to ensure that it was neutral in its formulation.

We utilized a flexible and dynamic participant-led approach to data collection, using prepared questions, but following the narrative of participants, paying attention to important contextual insights and social factors. Our interview approach used open-ended questions to allow participants to voice/frame their thoughts in their own fashion. Closed questions were used to verify understanding, clear up minor points and elicit information that was finite (e.g., age, education, professional status). We did not focus only on what individuals ‘knew’ about AMR and technical conducts on prescribing and dispensing AB; participants’ opinions, perspectives, personal and professional experiences were also explored within the core set of questions. The interview guide explored the following common sections to all health areas and specific content questions targeted to each health area; sections examples included general information about the health service and consultations, interaction between HPs and patients, practices and criteria for diagnosing, treatment option, prescription and dispensing of critical antibiotics, impact of the Covid-19 pandemic on the health units and health practices, knowledge, attitudes, and other practices.

A common core of questions referring to clinical practices of prescribing and dispensing antibiotics was developed, around which individualized interview guides were designed for each category of health professional. For GPs, questions targeting use AB prescription for children, pregnant and elderly people in recurrent cases (ex.: urinary tract infection, upper-respiratory tract infections, etc.) were addressed to better understand their practice; GPs’ roles as health managers in the territory were specifically explored as well. As nurses play a central role in managing the PCU in various areas, their knowledge about the community and institutional practices, shared consultation with GPs and quality assessment of material control in the unit were further explored. Pharmacists were inquired specifically about technical procedures for controlling, managing and supplying stocks of ABs, as well as their relationships with nurses and GPs in the unit, and their roles as health educators in the community. For dentists, mechanical and chemical interventions in common cases in PC, AB pre-exposure prophylaxis use, use of sensitive technology and material in endodontist’s practice and follow-ups with patients were explored in depth. Veterinarians were inquired as to the specificities of the health service, workflow between PC and secondary and tertiary care levels in the Veterinary Unit, specific cases that ABs are used both for treating and preventing infections after invasive procedures, and the interrelationship between human and animal infections. The determination of data saturation [19] was tied to the common core of questions. For that reason, there are differences in the number of interviewees across health areas. Given the specificity of the health settings, and, more importantly, the roles of each health profession in the context of prescribing and dispensing ABs, a minimum of twenty interviews were predetermined for this exploratory and localized study; however, given the different health areas, it was necessary to expand the number of participants to further explore the core set of questions for each health profession

### Data analysis

With the participants’ consent, interviews were audio-recorded. Interview data were fully transcribed. The audio recordings and the transcripts were pseudonymised by assigning a participant identification number to each interview.

### Coding the interview transcript

Line-by-line analysis of each transcript involved categorizing thematic parses of text referred to as *quotes* (e.g., a phrase, sentence or group of sentences conveying meaning). For each domain, the quotes with a shared underlying meaning were then summarised into a specific code. Excerpts presented in this article were translated from Portuguese by the authors. Field notes were taken during visits and/or immediately after interviews. These materials were then subject to thematic analysis [20–22].

### Reliability of coding and coding scheme

The first author independently annotated all interviews. The third and fourth authors collaborated in the creation and refinement of codes. A coding scheme was developed based on the initial data analysis, which then guided the analysis of the remaining transcripts. Discussions on coding were carried out with the research group throughout the project, ensuring consistency through the different areas of the study.

### Generating specific themes

Collections of responses with a similar underlying theme suggesting a problem and/or influence of a belief on health practice were identified as **specific themes**. Strong evidence for these specific themes must have been mentioned by the majority of the participants in HP category. Quotes are anonymized and participant IDs reflect their profession.

### Generating major themes

Interviews were then reviewed to understand the ways interviewees framed their practices and AMR in order to develop **key themes**. These themes were structured separately and then grouped into major clusters, as explored in the results and discussion sections that follow.

### Derivation of themes

Themes were derived from the data. Although the interview guide provided core areas of exploration, presenting themes discussed in the literature, minor and major themes were derived from the analysis process. Participants provided insightful and detailed information besides their everyday health practice, reporting the organization of health services and structural issues.

Data analysis was undertaken between March and July 2022.

### Techniques to enhance trustworthiness

As part of large qualitative study exploring the production of AMR in Brazil through social science perspectives, the investigators collaborated in all phases of the study, assessing the methods, data, analytical process, conclusions and reflections. Senior researchers validated all phases of the research. Interim results were further presented to an international group of multidisciplinary scholars studying AMR, who provided feedback and considerations for further exploration of the data.

## Results

In total, 25 interviews were conducted (5 dentists, 8 General Practitioners, 4 Pharmacists, 3 Nurses and 5 Veterinarians), for whom 18 (72%) self-identified as female, and 15 (60%) held a graduate specialization title in the health area. Participants’ ages ranged from 22 to 52 years. The average age of all participants was 35 (SD = 6.2). Aggregated age averages by health profession are as follows: dentists (46; SD = 6), GP (34; SD = 2.7), nurses (40; SD = 0.5), pharmacists (33; SD = 2.5) and veterinarians (26; SD = 2.1).

We identified three overlapping social drivers across HPs’ discourses on prescribing and dispensing ABs: individual and behavioral challenges; relational and contextual factors influencing the overprescription of ABs; and structural barriers and systemic contradictions in the SUS. The major thematic categories identified through our analyses are presented in Table 1. Additionally, we present a diagram of in-locus factors influencing AB prescribing and dispensing practices, discussing how they relate with systemic contradictions in PC. Finally, we discuss how societal multilevel factors intersect and are intrinsically intertwined with one another amid AB overprescription practices and AMR in PC.

**Table 1:** Themes and subthemes stemming from the analysis of the interviews.

**Individual and Behavioral Challenges**	Clinical and Health Practice	Uncertainty on deciding diagnosis and treatment
		Professional’s fear of (non)prescribing ABs
		Degree of awareness of AMR arising from AB overprescription
		Managing risks and danger: HP’s accountability
		ABs given as a preventive strategy
	Cognitive and knowledge attributes	Difficulty setting criteria for diagnosis and treatment
		Unawareness of infections transmitted between animals and humans in the context of AMR
		Professional experience as a major determinant for deciding treatment
		Doubts and lack of knowledge on adequate and critical classes of ABs
	Behavioral attributes	“Sovereign” clinic perspective as influence on diagnosing
		Individual ’style’ and HP’s personal beliefs on AB prescription
		Insecurity about clinical conduct
		Mediating practice, experience and knowledge of diagnosing and treatment
		Influence of previous education and workplaces on treatment with ABs
**Contextual and relational factors**	Social context and territory	Poverty and socio-economic vulnerability
		Lack of basic sanitary conditions in favelas and human occupied areas
		High prevalence of physical pain, violence and mental health issues in the territory
		Difficulty in reaching an Emergency Room in Hospital in some communities
		Territory is unequal and socio-economically heterogeneous
	HP and patient communication	Lack of negotiation on AB prescriptions
		AB self-medication by patients
		Demands for pediatric AB prescriptions
		Ineffective communication about AMR with patients
	Communication between HP	Lack of communication about AB overprescription and AMR
		Lack of coordinated actions between health teams in PCU
		Lack of time to discuss critical points and conduct
		Misunderstandings of AB prescription patterns between HPs
		Aggravated lack of communication between HPs during Covid-19 pandemic due to high workload
**Structural and health system barriers**	Functioning of the health system in PC	Contradictions on longitudinal and emergency care in PC
		Very heavy bureaucratic workload
		Poor working conditions (particularly for GPs, Nurses and Veterinarians)
		Lack of timely appointments and follow-ups
		Diverse views on whether primary care clinics should allow walk-ins
	Health system and resources	Lack of timely laboratorial and image exams
		HP’s hesitation in asking for antibiograms
		Lack of free medications for humans and no free veterinary pharmacy
		Lack of communication between health care levels in the city
	Territorial demands affecting health practices	HPs’ conduct is adapted to local demands
		HP tendency to prescribe more ABs amid poor local sanitization and precarious hygiene of patients
		Poor housing conditions that facilitate spread of infectious diseases
		Presence of the interaction between animal andhuman on AB use and AMR
		Lack of awareness by GPs of bacterial diseases that are transmitted from animals to humans

### Individual and behavioral challenges: Dismantling the security of knowledge

Inadequate practices towards prescribing and dispensing ABs amid the AMR threat extend beyond a lack of knowledge and education. HPs combine different diagnostic and treatment strategies that rely on individual cognitive and behavioral attributes, particularly related to one’s clinical practice and professional experience, together with knowledge of specific clinical practices. In this, HPs dismantled the idea that more knowledge leads to better clinical practices. However, there were key factors that fueled a professional’s ability to better decide on diagnostic criteria and lines of treatment. Gaps in knowledge and lack of in-locus resources increased uncertainty about diagnosing, while unawareness of adequate options of treatment led professionals to feel insecure, frequently resulting in the overprescription of AB. While professionals demonstrated knowledge about the risks and threats of inadequate AB prescribing patterns, they tended to work with diagnostic criteria and lines of treatment that, contradictorily, may lead to enhanced negative outcomes (ex.: subjective diagnostic criteria, excessive use of AB) and increased AMR.

Health professionals sometimes altered their perception of scientific evidence and efficacy based on clinical practice. A dentistry informant acknowledged that although he had learned that prescribing antibiotics was only recommended in the presence of fever and abscess, *‘I don’t follow this protocol*, *because I think that [if] the patient has already reached a very acute stage [without fever or abscesses]*, *we don’t need to [wait for the patient] to reach that point [in order to prescribe AB]’* (Dentist, 4). On the other hand, a GP reported that experience led her to take a more nuanced approach to prescribing AB, asserting that at an earlier stage of her career she *‘would put everyone on antibiotics’* and now she acts differently (GP, 10). In situations in which prescribers face uncertainty about the patient’s access to follow-up consultation and/or a lack of complementary exams, they *feared* that the disease of their patient, human or animal, would advance to a severe condition. This individual perception often led to AB overprescription: *‘I think we use them [antibiotics] without even knowing why*, *out of fear’* (Veterinarian, 22). Furthermore, a GP informant suggested that clinical practice provides the imperative to doubt scientific based practices as to whether to prescribe AB or not:

*Sometimes we hear infectious disease specialists saying*, *“don’t give antibiotics for a viral infection”*, *ok*, *but when you are on the frontline and you see that a patient is not doing well*, *[and] you don’t know which way to shoot anymore*, *right*? *Is it going to be that bad*? *To give antibiotics or not*? (GP, 6)

Health professionals tended to see their practice as a type of *‘sovereign clinic’* (GP, 10) and sometimes decided to diminish the number of antibiogram and image exams ordered; this happened partially in response to local constraints, such as the unavailability of exams in their units and the difficulty patients face in obtaining a timely exam appointment. These occurrences tended to become entangled in HPs’ prescribing patterns; additionally, given the lack of communication about AB overprescription and AMR in PC, these patterns tended to be maintained overtime as if the practices were a *personal attribute*; as this pharmacist states: ***‘****he [GP] is already used to that medication*, *so he just gives that [*…*] he prescribes the same thing for all patients*, *all day’* (Pharmacist, 19), saying that no matter how diverse the situations are, GPs tend to prescribe according to their *personality* and *habit*.

Health professionals’ perception of risk and danger were strongly related to their self-confidence on deciding a line of treatment, sometimes resulting in greater risks to patients. As this dentist informant pointed out, *‘you also start [acting] not wanting to take too much risk*. *Depending on the case*, *I give antibiotics right away’* (Dentist, 3). The risks HPs perceive may not correspond to the real risks and danger that patients experience (due to inadequate AB prescription); this results in a doctor-centered rationale of risk rather than one centered on the patient’s health. The risk management landscape seems complex to negotiate and to base decisions on and, as this GP asserted, defining major criteria for prescribing AB on respiratory tract infections is *‘the big question worth a million [dollars] in relation to the context we live in’* (GP, 7). While some HPs faced uncertainty due to the wide scope of risks, others minimized it:

*We are not allowed to prescribe AB for wounds*, *but it is something that I would feel confident*, *[in doing]*, *because I know the appearance of the secretion*, *I know how to see when there is a phlogistic sign*, *and I can assess whether he needs an antibiotic or not; we look at it*, *it’s right away*! *Today a man came in with a secretion on his leg*. *It was very red—automatic*!*—you squint*, *you know he needs to take an antibiotic*, *right*? (Nurse, 16)

Regarding decisions over risks and practices, HPs are faced with conflicting relational and contextual barriers in the health system, effectively resulting in layers of complexity in their practices and in enormous challenges both to improve adequate AB prescribing practices and change behavioral patterns.

### Contextual and relational factors: Catalyzers of antibiotic overprescription

Many HPs work in vulnerable socioeconomic areas where their human patients may lack access to basic sanitation infrastructure and struggle with conflicting social situations of violence, domestic abuse, and mental health issues. In the case of veterinary care, animals commonly arrived at the veterinary clinic with aggravated skin and advanced canine periodontal disease, in this way reflecting the lack of adequate hygiene practices. Interactions between humans and domestic animals, mostly dogs and cats, reveal the disparity between the contextual and environmental reality (e.g.: hygiene practices and infection dissemination in the territory). As a pharmacist reported, *‘human patients with wounds on their foot or leg [who have] a porous floor in their homes*, *which is not washable*, *[find that the sanitary conditions] contribute to worsening the infection*, *together with the fact that many patients lack the resources to have home caregivers’* to help them with hygiene practices at home (Pharmacist, 18). Additionally, GPs report that patients, without any bandage protection, play with their animals on top of their wounds. Antimicrobial resistance in chronic wounds were very often reported in the area:

*I think we end up treating this issue of wounds very empirically and we have many cases of patients with chronic wounds*, *which we treat once*, *twice*, *three*, *four times and then when you go to ask for the secretion culture*, *it is resistant to everything [*…*] also because of these social conditions*, *hygiene conditions that are sometimes not the best*, *so I think this is perhaps something for us to rethink* (GP, 9).

Accordingly, these contextual situations sometimes led prescribers to overprescribe ABs as a precautionary strategy:

*‘[*…*] we are even afraid of not starting the treatment with antibiotics*, *then the patient gets worse*, *and we do not have that control anymore’* (Veterinarian, 22).

Most of the animals’ owners hardly ever return to the unit due to having to wait many hours to be seen by a veterinarian. Similarly, GPs tend to decide whether to prescribe AB considering the contextual reality of the patients:

*I made a suture on a patient’s hand; we could see that the hands were very dirty*, *with dirty nails*, *it may not have any infection in there*, *but if I do not give an AB…*, *it evolves to an infection that I do not know [what it would be]*, *so sometimes at this point I would rather go on with the AB prescription than waiting to see what happens… It takes a while for them to come again*, *then something worse happens*. (GP, 11)

At the relational level, HPs experience a lack of communication about AB prescription and AMR in their human and animal health units. Although professionals demonstrated AMR awareness, the topic remained undiscussed in formal and informal meetings amongst HPs. Communication barriers, combined with a lack of discussion of clinical cases and common conduct guidelines, apparently reinforced individual prescribing behavior patterns. A nursing informant mentioned that every GP adopted a different clinical conduct, and because of high GP turnover in the unit, *‘the conduct [with the patient] keeps changing all the time*, *you know*, *I think this is bad for the patient*, *very bad’* (Nurse, 16). Moreover, GPs often maintained patterns of AB prescribing practices learned in different health care levels that were not adjusted to PC needs. For example, when GPs write insufficient patient information or inadequacies in prescribing AB, such as incorrect name or dosage, or when AB are not available in the pharmacy, *‘I can say [what the GP says for patients]is wrong in the pharmacy and the GP says it is right for the patient or vice versa*, *we cannot work with uncertainty*, *I want to clear up their [GPs’] doubts’* (Pharmacist, 18). Considering that lack of communication contributes to the invisibility of the problem and the absence of coordinated actions, problems with communication appeared to produce more conflicting demands amongst HPs.

Communication with patients about the risk of increasing AMR due to the misuse of ABs was often reported as limited and inefficient; In doctor-patient interactions, GPs asserted that very few patients understand AMR. Furthermore, human patients and animals’ owners sometimes used different primary and secondary health services for the same demand without disclosing their prescription record to GPs and veterinarians; this often resulted in the overprescription of ABs because there is no single electronic medical record for humans or animals in Brazil. In the city of São Paulo, pharmacists of the public pharmacies are the only professionals with access to the AB dispensing records of human patients. One participant reported a typical conversation conducted with a user: *“Look*, *did you tell the doctor that you already took this antibiotic at the beginning of the month*, *and you are taking it again*?*” and the patients tend to provide answers such as*, *“No*, *I did not*, *he is aware [of it] and he asked me to take it a little longer” […] it is difficult to know if they really spoke*, *or if they did not”* (Pharmacist, 17). This scenario reveals that communication, both between HPs and between professionals and patients, was considered inefficient in regards to problems of overprescription and misuse of AB, thus catalyzing negative health consequences in PC.

### Health service operations, resources and territorial demands: Systemic contradictions and ambivalences

Health professionals navigate through structural factors in PC: (i) inadequate access to health care services, high demand and management issues; (ii) bureaucracy, lack of resources (exams and medications); and (iii) multitasking working structure and exhausting working conditions. These structural barriers play a central role in how HPs responded to their work demands and, more importantly, mediated their judgment on diagnosing and deciding lines of treatment. As underlying factors, contradictions were identified in the system; for example, prescribing AB as a *precautionary strategy* to attempt to mitigate structural barriers due to insufficient number of follow-ups and health resources. Additionally, AB prescribers reported ambivalences; while HPs were aware of risks about AB overprescription and AMR, they affirmed feeling *‘impotent’* (GP, 9) in not being able to provide adequate responses to patients, due mostly to how the health system is structured and operates.

The high demand for services at the health units leads HPs to prescribe antibiotics in the first consultation, partially because of the uncertainty about whether and when follow-up appointments will occur. In the words of a dentist, *‘It is better to give an antibiotic*, *it is more often a prevention*, *even more here in the unit where we do not have frequent contact with the patient; it is for him to be calmer’* (Dentist, 4). GPs moreover warn that PC is absorbing demands from secondary and tertiary health care levels, increasing the number of appointments for urgency and emergency in the unit: *‘primary care is kind of turning into an Emergency Room’* (GP, 10). This GP stated that the increase in workload in PC was greater than in an Emergency Room, because the patient ultimately is *‘ours’; this refers to the fact that* the patient is registered in the PCU, and therefore, has the right to be monitored after the visit, *‘so I think it turned out to be a harder job than [taking care of] a crowded emergency room’* (GP, 10).

With multiple access paths to PC, health units lack necessary resources to take care of the number of human patients seeking services; for example, the lack of ABs in the free public health pharmacy directly affects a GP’s clinical conduct. Knowing the patients may not be able to afford the prices at private pharmacies, GPs prescribe the AB that is available in the free pharmacy regardless of its AB class. Moreover, the unavailability of on-time laboratory and image exams was often reported as a major factor affecting the quality of the human and animal diagnoses, particularly in respiratory and urinary tract infections:

*So*, *when is the right time to put an elderly person on antibiotics if I do not have an X-ray here [*…*] you do not necessarily find a focus [of infection] and then you start or do not start with antibiotics*? *[*…*] so*, *what is the moment I anticipate [the prescription] to try not to make the problem serious or I will be [making a mistake]*? *Maybe this is the great bias here*. (GP, 7)

*I believe that if*, *for example*, *there were more types of tests for us to confirm [the diagnosis]*, *we would not get our hands on antibiotics so quickly [*…*] Because sometimes we schedule an ultrasound*, *“Ah*, *the ultrasound is only [available] within 20 days now”*, *and the pet won’t be able to wait 20 days*, *so there’s this issue too*. (Veterinarian, 23)

HPs for human health are considered *care managers* in the SUS. A GP reported that bureaucratic work negatively affects their ability to listen carefully to the patient and provide a proper diagnosis and treatment strategy (GP, 8). In addition to insufficient resources, bureaucratic work demands much of the HPs’ time (especially Nurses, GPs and Pharmacists), diminishing their time for consultations and activities in the community. For example, GPs reported having only 15 minutes for a regular appointment, but still must fill out and revise several forms during the consultation. HPs reported that bureaucratic work with high demand and workload contribute to in the poor working conditions. They also disclosed that these working conditions negatively impacted both their mental health and their ability to manage and mediate several simultaneous tasks. As a GP summarized: ‘*there were GPs who came in and stayed for a week and then quit*, *so I think the work is very hard*, *you know*, *I think it impacts [on our health] a lot*, *because I think many people [GP] get sick in the [working] process*’ (GP, 8).

These practices all show that, to one degree or another, HPs must have the ability to mediate structural barriers and deal with contextual, relational and interpersonal factors, while engaging in appropriate diagnostic and AB prescription practices. Given the lack of guidelines, One Health awareness and protocol practices in PC, the HPs themselves must assess the factors to be considered crucial in determining the appropriate prescribing pathways.

**Fig 1:**
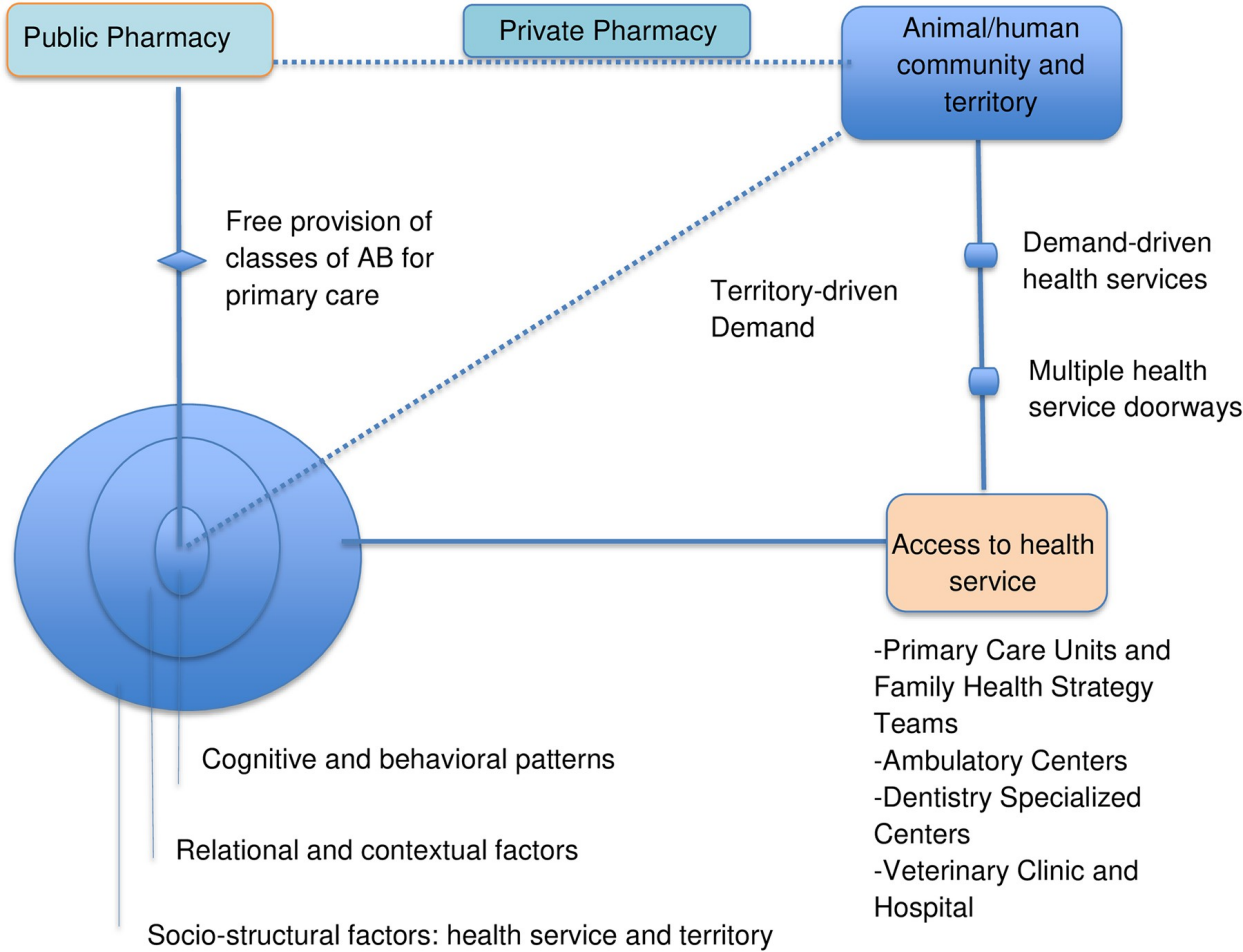
Diagram of in-locus overlapping factors informing prescribing and dispensing practices in primary care. This diagram presents all major health settings that are available for human and animal patients in primary care, including the community and territorial demands for health units. In São Paulo, human medicine disposal procedures, vaccination and epidemiological surveillance are also activities PCUs oversee in their coverage area. Accessing the health service, both the human and animal sectors patients are sent to AB prescribers without sorting their needs. Public pharmacies for human health provide only essential medications, including classes of AB, as part of a national and local intervention, controlling the availability of ABs for patients in public services. Medications unavailable in the public pharmacy may be purchased in private pharmacies or veterinary clinics and stores. There are several types of services for human health in primary and secondary health care levels in the community; in certain cases, patients may be confused regarding the service most appropriate for them.

We propose a diagram to represent the interactions of in-locus factors related to AB prescription (Fig 1), which presents all the major health settings that patients can use in primary care. We attempt to locate these health settings within the community these patients live and the territorial demands in the area. Thus, Fig 1 shows the major elements informing HPs’ practices on diagnosing, treatment and dispensing practices that we found to be major social drivers. Our findings indicate that prescribers cope with three layers of factors (individual, contextual and relational, and structural dimensions) that are dynamically intertwined in practice, exerting varying degrees of influence on diagnosis and treatment.

While territorial demands and pharmacy services are all connected in order to provide AB approved by the SUS for human patients, animal health has a different system, which does not offer free medication. The extension of services, actions and demands from the territory indicates the responsibility of the health system and professionals in coping with barriers, challenges and contradictions at every service level. In this way, Table 2 presents key areas for interventions that HPs raised, accounting for the local community, health services and possible national interventions.

**Table 2:** Potential interventions in critical areas raised by human and animal health professionals.

Primary Care level interventions	Public Health interventions
• Increase human and material capacities to attend patients in primary care; • Establish real separation between longitudinal and urgency/emergency care services; • Triage patients’ needs prior to appointment with general practitioners (particularly for walk-in units [advanced access]); • Provide training/education to health professionals on AB prescribing practices and antimicrobial resistance; • Provide complementary ready-to-use materials orienting antibiotic prescription practices; • Establish ‘dedicated time’ for communication between health professionals on antimicrobial resistance; • Create structures to receive feedback on prescribing patterns in primary care; • Set coordination areas on antimicrobial resistance and antibiotic prescribing practices in the territory/region or in the health unit; • Increase ability of the primary care Health Units to manage medication residues from the unit and territory.	• Increase availability of timely laboratory and image exams to human and animal health services (particularly for Veterinary Clinics and primary care Health Units); • Create a surveillance system to notify cases of antimicrobial resistance in primary care; • Increase availability of antibiotics in the free public pharmacies, particularly in very vulnerable socioeconomic areas; • Increase logistics management and processes to dispense exact amount of antibiotic to patients; • Increase capacity and training of health professionals for material quality control in the primary care units; • Create free public pharmacies for Veterinary use; • Create educational interventions for community implementation on topics of antibiotic use and antimicrobial resistance; • Extend to the community educational interventions on topics of hygiene and human-animal zoonosis; • Create educational interventions for community implementation on topics of animal health (domestic medication); • Create educational interventions for community implementation on the services individuals should use, based on their needs; • Create taskforce in collaboration with municipal level participation to provide or improve basic sanitization in vulnerable areas.

We separated primary care and public health interventions into two categories, although we see a clear overlap between them.

## Discussion

Our findings indicate the influence of multilevel factors and social drivers affecting AB prescription and dispensing practices for humans and animals, highlighting the extension of social and sanitary problems in PC. Although AB overprescription is widely attributed to prescriber’s behavior and knowledge [2, 3, 23], we provide evidence that HPs experience contextual and territorial challenges that directly influence their risk perception, diagnosis, use of laboratorial and image exams, time and decision to undergo treatment, choice of Abs to prescribe and coping strategies on AB prescriptions. Our findings parallel current global evidence [6, 24, 25] on AB prescribing and dispensing practices being shaped by acute socio-economic issues at the local level, along with infrastructural and health access issues in human and animal health systems,. While socio-economic deprivation in housing, environment and work affects the quality of health from *in utero* to older age [26], we found that antibiotics are seen as *remedial* in contexts where patients have difficulty accessing the health system, live in poor sanitary and housing conditions, and have scarce knowledge/practice of hygiene practices. Thus, prescribers in PC cope with multilevel social deprivation and biomedical factors [6, 24, 27], aggravated further by strenuous working conditions and extensive bureaucratic work in PC.

These findings suggest that simply increasing awareness and knowledge of AMR, without tackling in-locus challenges and systemic contradictions, including social ambivalences, are insufficient to change AB prescribing practices. Specialized literature has provided evidence that ABs are used at times as a *band-aids* to mitigate socio-economic disparities to which patients are subject, together with providing care to vulnerable populations lacking basic health resources [6]. While these strategies are intrinsically connected to AB clinical effects, they rely on AB’s symbolic value shared between HPs and patients in the community [6, 28]. Alternatively, HPs in our study reported that when ABs are used pragmatically in situations in which the prescribers’ ability to follow up with patients is restricted, prescribers tend to feel they are *on the safe side* due to the perception that ABs mitigate potential harmful risks to patients. More importantly, these circumstances mostly occur in the presence of lack of health resources and unavailability of consultations. Similarly, these Brazilians findings of AB overprescription prescribing practices are strongly associated with socio-economic determinants, particularly in LMICs [23, 25, 29]. We further found that while prescribers recognize their practical role in mitigating negative risks to patients, their practices nevertheless add layers of unmeasurable risk to patients in trying to cope with health system issues. In this scenario, prescribers may cope with ambivalences amid such risk management: while knowing the hazards of AB overprescription, these are counterbalanced by the reality of the patient’s situation and needs (concrete concerns). This leads to an individual physician’s cognitive/concrete ambivalence. In addition, while some HPs claim the ‘sovereign clinic’ as their clinical practice, others are ambivalent about the necessity to add more resources to help with diagnosis and treatment.

Dentists and veterinarians frequently report these ambivalences in their practices. There is currently a vigorous debate on whether dentists should prescribe ABs prior invasive interventions in at-risk patients with endocarditis [30–32]; we found the spectrum of AB prophylactic use extended in PC, both prior to and after pulpitis and canal interventions. Considering the HPs’ awareness about the risks of AB overprescription and AMR, AB prophylactic use is one of the coping strategies dentists adopt to manage the ambivalence of responding to concrete vulnerable situations [32], producing new layers of risks to patients and AMR. These findings align with a few other studies indicating that AB overprescription and/or AB prescriptions are mostly based on professional experience rather than sufficient scientific evidence, in dentistry practices across countries [32, 33]. Conversely, veterinary practitioners mostly receive patients in very poor health who are using ABs acquired without a prescription. Noting that laboratorial resources are scarce, practitioners tend to prescribe AB immediately in an attempt to meliorate patient’s evolution. Veterinarians described this as their *out-of-fear* motivated practice, indicating that the HP’s final decision on whether prescribing AB and when is mostly affected by potential imminent danger rather than long-term risks [3]; knowledge gaps and prescribing practices without oversight are additional issues observed in this context [34]. Thus, the risk-managing landscape responding to these ambivalences reinforces the argument that AMR responses in public health fail due to these *quick-fix* actions and temporal myopia in interventions [3, 10, 13].

The local reality we explored, however, is extremely dynamic and presents challenges to HPs towards controlling the duration and setbacks of health issues. Noting that PC aims to provide longitudinal care, HPs report an increase in emergency consultations amid extensive workload related to attending both regular and emergency cases. Teixeira Rodrigues et al. (2021) have indicated that physicians’ knowledge and attitudes regarding AB prescribing differ from hospital to PC mostly due to the clinical practice in each [35]. These situations challenge the prescriber’s abilities at work, and the idea that the GPs themselves are responsible and trusted to give the best treatment across complex cases [36, 37] reaffirms an individual economy of risk over the durability and setbacks of the health issues within the local context. More precisely, risks are socially produced and economic unequally distributed [38, 39]. These risks are tackled individually in PC, thus increasing the burden of responsibility on the role of prescribers [36, 40]. This indicates that while HP’s decision in this context may reflect a shared *habitus* contextual health practice [41], an individual-societal balancing factors [4], or an experiential knowledge practice [42], ABs are more likely to be used as a *material remedial* in the economy of AMR risks.

Noting that an individual economy of risk may enhance prescribers’ power to influence outcomes associated with AMR, GPs are moreover central players as health managers for human health in the SUS. Interestingly, HPs reported no surveillance and targeted actions to prevent AB overprescription and AMR cases in human and animal PC; corroborating recent evidence from other LMICs [23, 29], this reveals a neglected work-function accompanied by overprescription of AB without oversight. In Brazil, the lack of free essential AB provision in public pharmacies is a constraining factor in this dynamic [43], leading human health prescribers to change their prescription to guarantee that patients have access to the medications available in the public pharmacy.

As nurses play a central role in PC, sharing prescribing responsibilities with GPs is part of a large ongoing debate about health policies, educational and professional attributes in nursing practices [44, 45]. In our study, nurses’ prescribing practices are limited to a few established national protocols in PC. While some nursing practitioners may be unaware of the circumstances under which they can prescribe ABs, their discourse is aligned with indications of the necessity to advance with interprofessional collaboration on AB prescriptions in PC [44, 46]. Considering the importance of HPs in local health management, evidence in high-income and LMICs’ countries suggests AB prescribers struggle with lack of knowledge, skills, professional experience and self-efficacy in order to perform their work as clinicians [23–25, 35].

These circumstances may be enhanced due to the lack of effective communication on AB prescription and AMR between HPs amongst themselves and with patients. While the lack of effective communication within health services is associated with overall negative health outcomes [47], we found it may play a substantial role in catalyzing negative outcomes with respect to AMR across human and animal health areas. We pointed out that while HPs experience difficulties in having practice-oriented communication, AB prescription patterns constitute a fundamental element that reinforces prescribers’ own *personal style* and *habits* regarding AB prescription. The lack or minimization of communication efforts in health practice is associated with higher AB prescription across countries [48, 49]. Noting that HPs’ risk perception, knowledge and strategies towards diagnosis and treatment with AB are not shared amongst themselves, ABs are more likely to be used inappropriately [49], such as in pre- and post-invasive dental treatments [27]. Similarly, observing imprecisions and misconducts on prescriptions for humans, studies have indicated that pharmacists can act both as gatekeepers and health educators to prevent inadequate types and quantities of ABs provided to patients [50]. Finally, the local lack of systemic communication between human health service levels, public and private health units, together with nonexistent single patient’s record constitute an enormous barrier towards controlling AB overprescription in Brazil.

How can the problems related to securing best practices on AMR and AB use be addressed in the SUS? Some issues have been explored from different perspectives in the literature, pointing out the centrality of the Health System and the necessity of addressing political and practical responses as a structural factor within health interventions [51, 52]. The *health structure* has been viewed both as a frame to organize policy, protocols and services, and as a dynamic framework that receives bi-directional inputs from health services and stakeholders [53]. As presented in **Fig 1**, many of the key factors determining AB prescription and dispensing practices inherently overlap: they are mutually co-produced and simultaneously occur at different micro-levels within the operational structure of PC. Additionally, these key factors are profoundly shaped by risks and perception of risks subject to human management; these social-biomedical factors lay open to the social process of definition precisely because of their human determination and social action [38]. While specialized literature has focused on specific, individual and behavioral change interventions [3, 10, 11], we suggest that drawing on the local and social possibilities of these socially open processes of definition may produce better health outcomes to patients in their communities [11, 52, 54, 55]. This approach may guide the organization of t the work and practices of HPs towards optimal long-term reproducibility of adequate AB prescription stewardship practices in PC [13]. A concern with meaningful, effective and successful interventions leads us to consider how we can make interventions that do not run counter to the many existing frameworks and practices in PC. While HPs suggested practical interventions in order to address the problem of AB prescription practices and AMR (Table 2), those types of interventions require implementation research designed in accordance with efficient integrated health actions in PC [14, 56].

To our knowledge, this research was a pioneering One Health perspective study with diverse HP in PC in Brazil, with the aim of comprehensively exploring broad views and perspectives regarding AB prescribing and dispensing practices across health areas. This study’s design and results present limitations, as the specificities of each health area within the context of PC regarding AB prescriptions and dispensing practices merit further investigation. We point out that our results are contextualized to an urban setting in an extremely socio-economic unequal city, while regional, urban and rural differences in Brazil are preponderant. Additionally, as many HPs were unaware of the One Health perspective, they presented scarce knowledge about the relationship between human, animal and environment as major health determinants on AMR, a fact that limited our exploration about the theme and consideration of possible interventions tackling those problems together in PC. This research focused on exploring trained HP practices and views through a relational perspective and did not capture awareness of AMR or practices of AB prescribing and dispensing among private sector providers. Although most human antimicrobial use is likely to occur with people using the public National Health System, our findings of multilevel factors affecting health practice may not resonate with the private sector, particularly with veterinary and dentistry practitioners. Additionally, sampling for each health area varied reflecting their roles and context in the health services: at the time of data collection, we interviewed all GPs and pharmacists working in the PCU (our primary setting of exploration) and AHS, respectively; 33% of nurses were interviewed in the PCU in the Health Family Teams (3/10), 83% of dentists (2/2 working in PC and 3/4 working in specialized care) and 83% of general practice veterinarians participated in the study (5/6; the overall number of veterinarians working in all areas of the Veterinary Hospital/Clinic is 35).

## Conclusion

Our study aimed to explore the social drivers related to inappropriate prescriptions of AB, presenting a discussion of the social implications of this increasing public health threat in the context of PC of human and animal health in a Brazilian city. Through a relational One Health perspective between social drivers affecting AMR and PC we identified that in-locus factors influencing antibiotic prescriptions and dispensing practices are intertwined with individual accounts of risk management, systemic contradictions and ambivalences in the national health system. Qualitative studies offer not only pathways for further examination extrapolating certain contexts, but they also reveal the pervasiveness of distal and structural social relations affecting microlevel activities. This approach allowed us to reflect on the complexities involving socially deprived populations, the social production of risks and the challenges health systems face in responding to the increasing global threat of AMR.
